# Disease insights from brain somatic mosaicism

**DOI:** 10.1038/s12276-024-01331-x

**Published:** 2026-04-08

**Authors:** Changuk Chung, Rahul Nedunuri, Joseph G. Gleeson

**Affiliations:** 1 Department of Neurosciences, University of California San Diego, La Jolla, CA 92037 USA; 2 Rady Children’s Institute for Genomic Medicine, San Diego, CA 92123 USA

**Keywords:** Genetics, Neurological disorders, Genetics research

## Abstract

Brain somatic mosaicism (BSM) refers to genome variation within brain cells that results from accumulated postzygotic mutations. These mutations can be used to understand cell lineage, molecular dynamics and disease processes. Unlike most other organs, brain cells are mostly fixed in position and not replaced throughout life. Thus, assessing mosaic variants (MVs) within the brain, including their spread and cell type-specific distributions and correlations with aging and cellular health, can reveal insights into neurodevelopmental, neuropsychiatric and neurodegenerative diseases. Extracting genetic material from human surgical brain resections, pregnancy remnants, or postmortem samples can reveal the origins of brain cells and uncover the effects of aging and disease on genomic integrity. Technological advances combining high-read-depth bulk sequencing, isolation of specific brain cell types, and single-cell multiomics can both detect and quantify MVs with good precision and recall. Research exploiting brain MVs is revolutionizing the understanding of the origins, mechanisms and potential treatments for brain conditions.

## Brain Mosaicism Unveiled: Insights into Neurological Diseases and Aging

Our bodies consist of cells carrying genetic material, which can alter over time due to mutations. These changes, called mosaic variants, are studied to understand their impact on the brain. Some MVs can cause diseases like cancer, while others are neutral and help trace brain cell development. Researchers focused on examining these mutations in various brain cells, using advanced techniques to read genetic information. This enabled tracing cell lineage, showing their relationships and migration within the brain. The study involved looking at genetic material from different brain cells to understand mutation patterns. The findings showed that MVs build up in brain cells over time, with specific patterns linked to aging and neurological diseases. Researchers concluded that studying these mutations offers valuable insights into brain development, aging, and disease. This summary was initially drafted using artificial intelligence, then revised and fact-checked by the author.

## Introduction

The genetic material within the cells of the human body is not uniform. Instead, genetic mutations accumulate during homeostasis and cell division and are faithfully propagated to daughter cells. These mutations are referred to as mosaic variants (MVs) because they are present in only a subset of cells. On average, a new MV arises at every cell division, thus marking the lineage of each founder cell through its clonal progeny. If a given MV is present in multiple cells within a pool, then we refer to it as ‘clonal’. In contrast, if the mutation is present in a single cell such that specialized methods are required for detection, then it is ‘nonclonal’.

Traditionally, MVs in certain genes, such as p53, have been linked to cancer, as they can change the proliferative potential of the cells in which it occurs^1^. However, most MVs within the brain are likely ‘neutral’ to cell behavior and thus can be repurposed as natural lineage markers that allow for the deconvolution of clonal relationships between cells within the human brain^2–6^. Using this analysis, it is possible to compare models of brain cell migration derived from animal models with experimental evidence in humans.

Technological advances have led to more sensitive and precise methods for detecting MVs, providing potential applications for clinical assessment^7–13^. Increasing evidence suggests that a subset of noncancer-related MVs are associated with both neurological and psychiatric conditions^14–18^. Whether these MVs are a cause or effect of these clinical conditions remains hotly debated. Here, we summarize the key concepts of MV origins, methods of detection, and strategies for interpretation.

## MVs during early embryonic proliferation versus neurogenesis

**Fig. 1:**
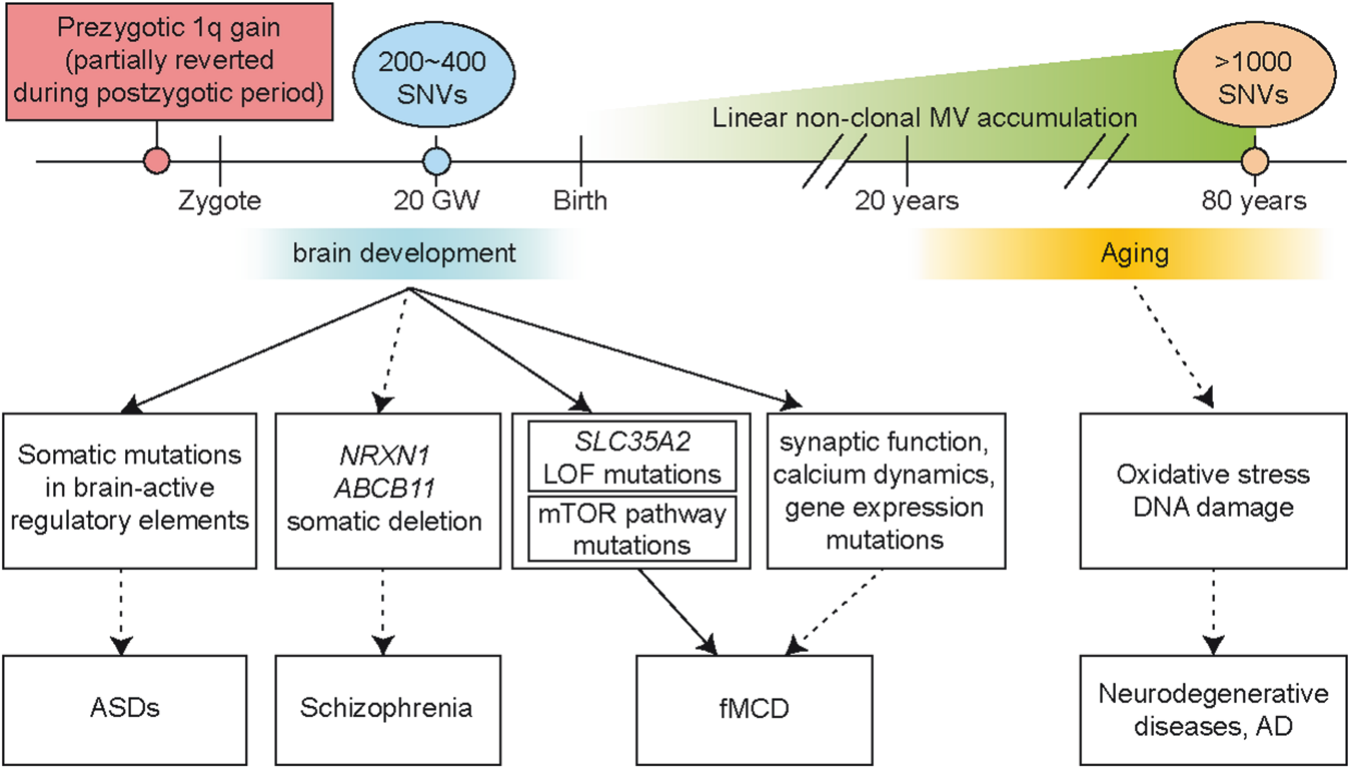
BSM is associated with human brain diseases throughout the lifespan. MVs in neurons linearly accumulate across the lifespan (ovals) in healthy brains and are associated with various brain diseases (white boxes). Clonal MVs generated in mitotic cells are associated with multiple neurodevelopmental disorders, such as focal malformations of cortical development (fMCD), autism spectrum disorders (ASDs) and schizophrenia. In contrast, nonclonal MVs acquired in postmitotic cells are implicated in neurodegenerative diseases such as Alzheimer’s disease (AD). Some MVs are derived from prezygotic germ cells and become mosaic via postzygotic partial revertant rescue (red box). Solid line, causal relationship; dashed lines, association; pcw, postconception weeks; mo, months.

The human brain comprises over 100 billion cells derived from a founder population of perhaps 50–200 committed neural precursor cells^3,4^. By gestational week 20 in humans, corresponding to the completion of neocortical neurogenesis, each neural progenitor cell carries between 200 and 400 single nucleotide variants (SNVs) at an average mutational rate of 5.1 SNVs per cell division (Fig. 1)^19^. This rate exceeds the approximately 1–3.5 SNVs per cell observed during early preneural embryogenesis^18,20–22^ and suggests vulnerability in neural precursors. The mutations observed in the developing human brain correspond primarily to the clock-like SBS1 signature found in the Catalogue of Somatic Mutations in Cancer (COSMIC), consisting of mostly C > T, varying at the bases surrounding the mutated base^23^. While the mutational pattern during early neurogenesis is attributed to the deamination of methylcytosine (mC) to T and corresponds to phases of rapid methylcytosine remodeling^21,24^, neurogenesis in later stages of development is associated with signatures related to oxidative damage^19^. Overall, the process of neurogenesis displays unique mutational patterns distinct from those observed in early embryonic proliferation, likely due to differences in the mechanisms driving division and epigenetic changes, as well as both cell-intrinsic and extrinsic factors.

## MVs acquired from postmitotic neurons

Neurons are examples of postmitotic cells that acquire MVs at relatively stable rates^8,15,25,26^ (Fig. 1). Because most neurons are likely terminally postmitotic, MVs that arise after exit from the cell cycle likely remain unique to each cell, without the potential to pass to daughter cells and are thus nonclonal. Detecting these mutations poses considerable challenges when using bulk sample sequencing because they are masked by the overwhelming presence of reads that match the germline. Fortunately, the advent of single-nuclei whole-genome amplification (snWGA) technology^27–29^ or single-molecule resolution sequencing with extremely low error rates^7,30^ has allowed the detection of nonclonal SNVs. It is estimated that approximately 16 SNVs and 3 insertions or deletions (indels) accumulate additively every year in the autosomes of human cortical postmitotic neurons. Thus, by the age of 80 years, each neuron is likely to carry >1000 SNVs (Fig. 1). These mutations accumulate during normal aging, impacting a tiny fraction of each cell’s genome.

While these estimates of mutation rates are widely accepted, it is difficult to reconcile the recent finding from the Brain Somatic Mosaicism Network that approximately 6% of neurotypical human cadavers exhibit orders of magnitude greater age-dependent MVs per cell^31^. Although the causes remain unknown, several of these individuals were found to have certain genetic or environmental risk factors that could have contributed to their ‘hypermutability’. Such individuals might be at greater risk for MV-associated brain conditions, but this hypothesis has not yet been tested.

Mature neurons present specific mutational types. Notably, SBS1, 5 and 16 accumulate linearly with age, suggesting that these mutational patterns are age-related in neurons^25,26^. Additionally, SBS5 and 16 exhibit a transcriptional strand bias^23^, implying that the clock-like accumulation of SNVs in mature neurons is associated with transcriptional activity. The other signature increasing with age resembles the COSMIC signatures associated with oxidative DNA damage. It increases moderately in normal neurons across age but dramatically increases in neurodegenerative conditions^15,25^. This difference suggests that moderate oxidative stress influences the build-up of SNVs throughout the lifespan but could be exacerbated by cellular stress in the form of neurodegenerative conditions such as Alzheimer’s disease (AD) (Fig. 1).

## MVs in glial cells

Compared with neurons, MVs within oligodendrocytes exhibit unique characteristics^8,26,32^. A recent study analyzed genomic variations across different ages in oligodendrocytes via single nucleus whole genome amplification (snWGA) combined with oligodendrocyte cell isolation^26^. Notably, SNV accumulation was 81% faster in oligodendrocytes (29 per year) than in neurons (21 per year), whereas the accumulation of indels was 28% slower (2.1 per year in oligodendrocytes vs. 2.9 per year in neurons). Moreover, the distribution of mutations differed, with MVs in neurons often found in transcriptionally active or brain-specific regulatory regions^8^. In contrast, MVs in oligodendrocytes were more common in genomically inactive regions^26^. These distinct MV patterns in glial cells compared with those in neurons suggest that distinct cellular processes influence MV acquisition. From a pathological perspective, patterns of MVs in oligodendrocytes strongly resemble those observed in glioblastoma (GBM). Given that MVs acquired in proliferative oligodendrocyte precursor cells (OPCs) are predicted to be transmitted to mature oligodendrocytes, this resemblance supports the hypothesis that OPCs may be the origin of the GBM. Further research is necessary to understand whether these MVs influence brain cancer, aging or neurodegenerative diseases.

## Mosaicism by prezygotic mutations and postzygotic reversion

BSM develops during the postzygotic phase, yet its somatic variations can also surprisingly stem from the prezygotic period if coupled with strong selective pressure. A notable example is revertant BSM in focal epilepsy^33^, where 1q tetrasomy, which originates from a maternal chromosome before fertilization and is presumably inherited by the zygote, is partially corrected in some embryonic cells (Fig. 1). This led to mosaicism of 1q tetrasomy in the brain, which is associated with focal cortical dysplasia (FCD) and intractable epilepsy, whereas other cells tested in the body lacked the 1q tetrasomy. Such somatic reversion suggests that certain mutations can undergo negative selection in some cell types but not all brain cells. The study further revealed that 1q tetrasomy is mostly specific to astrocytes, which seemingly tolerate this partial aneuploidy; however, the question of how cells correct aneuploidy remains unanswered^34^.

## Mosaic structural variants (SVs) in the human brain

While a clonal approach involving fetal human brains revealed no large-scale mosaic copy number variants (CNVs)^35^, in contrast, snWGA approaches involving postmortem adult brains revealed that 13 to 41% of the human frontal cortical neurons tested presented at least a single megabase-scale de novo CNV^36^. While still debated, the results suggest that megabase-scale CNVs in neurons could arise during later stages of development. Another concurrent study reported that many neurons contained one or more large candidate private CNVs, including at chromosome 15q13.2-13.3, a region frequently duplicated in neuropsychiatric conditions^37^.

Mobile genetic elements such as retrotransposons are sequences capable of transcription and translation, which then form ribonucleoprotein complexes with reverse transcription activity, creating complementary DNA that can integrate into new locations within the genome^38,39^. This process results in the formation of new mutagenic elements, introducing variability in repeat number and placement, thus contributing to BSM. In the developing brain, L1 retrotransposon activity within neuronal progenitor cells can alter neuronal functions, including gene expression and neuronal maturation^40^.

Moreover, L1 retrotransposons may contribute to the generation of CNVs. While L1 retrotransposons contribute to DNA damage, such as double-strand breaks^41,42^, and between 13% and 41% of neurons in the human frontal cortex exhibit large-scale de novo somatic CNVs^36^, direct evidence remains unproven. Intriguingly, L1 accumulation was inhibited during early life in mice by increasing maternal care, suggesting the potential for environmental factors to shape the genomic variability of individual brain cells^43^.

## MV barcode analysis: BSM as natural lineage markers

The human brain is predominantly composed of neurons and glial cells. Most of these brain cells are generated and migrate over varying distances to different brain regions during development, and while most of their progenitors are likely lineage restricted, some progenitors have the capacity to differentiate into several different brain cells. Moreover, since the human brain is highly complex compared with that of other mammals, lineage tracing in human brains may provide insight into the rules governing assembly and highlight human-specific or evolutionary features.

Traditional methods of tracing cellular lineages, such as dyes, viral tracers, or mutagenic barcodes in animal studies, are not easily applied to humans. Attempts to map lineage trees of human brain cells using either in vitro cultures of primary tissues or reprogrammed cells from human tissues have shed light on possible lineage relationships but have left questions about their relevance to in vivo biology. Recent advances in single-cell transcriptomics have allowed for pseudotime or RNA velocity analysis^44–49^, which can infer paths of development. Nonetheless, these approaches rely on the assumption that transcriptomic similarities accurately reflect lineage relationships, which may or may not be accurate.

Alternative methods that analyze neutral MVs in postmortem human brains, referred to as MV barcode analysis (MVBA), offer direct delineation of cellular lineage relationships within the mature neurotypical human brain. Just as a greater number of single nucleotide polymorphisms shared among individuals reflects a closer genetic relationship in population genetics, a greater number of shared MVs among progeny cells indicate a more common origin. Moreover, because neurons are postmitotic and likely do not migrate or replenish after maturity^50^, the pattern of MVs within the postmortem brain likely reflects embryonic origins, offering a retrospective view of developmental processes.

One of the early studies using MVBA utilized L1 elements, where WGS and subsequent PCR validation identified two retrotransposition events^51^. One was identified widely across several brain regions, suggesting that it arose early in development. The second was localized within the middle frontal gyrus, suggesting that it arose after lobar specification. Furthermore, by examining poly-A tail length variants, detailed spatial sublineages within the middle frontal gyrus emerged.

**Fig. 2:**
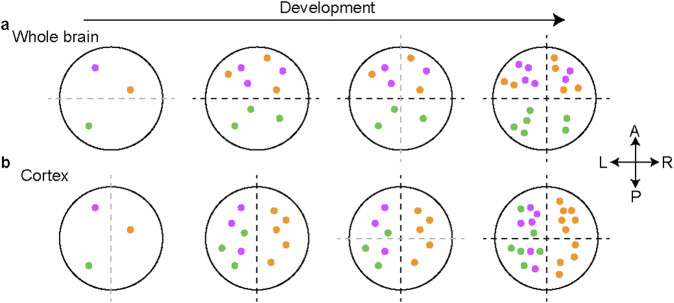
MV distribution reveals clonal restriction during human brain development. **a** The distribution patterns of MVs (colored dots) revealed that clonal restrictions between the forebrain and hindbrain are more pronounced than those along the midline axis are, which indicates that clonal restriction from anterior to posterior precedes the restriction from left to right across the entire brain. **b** Conversely, in the cortex, the pattern of clonal restriction differs: the left-right clonal boundary is more defined than the anterior-posterior boundary is, implying that clonal restriction along the midline occurs before the restriction along the anterior-posterior axis. The gray dashed line represents the emerging clonal restriction boundary, and the black dashed line represents the stronger restriction boundary than the gray dashed line. A, anterior; P, posterior; L, left; R, right.

Advances in techniques have improved the accuracy of MV detection for both SNVs and indels, especially for low allelic fraction MVs, which make up most variants. These breakthroughs have significantly enhanced clonal relationship resolution^2–6^^,52^^,53^. The combination of high-depth (250 ~ 300X) whole-genome sequencing, along with artificial intelligence-based improved MV detection algorithms and ultrahigh-read-depth amplicon sequencing used for both validation and precise measurements of allelic fractions, has revolutionized MVBA. With these approaches, we and others have recently clarified the clonal relationships between major organs of the body, estimated when the brain lobes become lineage restricted from one another, and calculated that human forebrain organogenesis initiates from a starting pool of approximately 90 to 200 founder cells^3,4^. This approach also revealed that while the human brain is clonally restricted along the anterior‒posterior axis, the cortex exhibits more significant clonal restriction along the left-right axis^3–5^ (Fig. 2).

Recent efforts have incorporated fluorescence-activated nuclei sorting (FANS) to isolate individual brain cell types on the basis of established epitopes, referred to as cell type-resolved MVBA or cMVBA. These methods can be coupled with single-nucleus RNA-Seq, WGS or even more recent protocols that allow for both RNA-Seq and WGS from single nuclei. These powerful methods have started to reveal the clonal distributions and dynamics of specific brain cell types, such as excitatory and inhibitory neurons, oligodendrocytes and microglia^2,6^^,53^. Huang et al. introduced parallel RNA and DNA analysis after deep sequencing (PRDD-Seq) to concurrently reconstruct neuronal cell types and cell lineages^53^. The authors were able to confirm early divergence of precursors for excitatory and inhibitory neurons and an “inside-out” layer formation of excitatory neurons, as documented in laboratory animals. FANS isolation of brain cell types (excitatory/inhibitory neurons, astrocytes, oligodendrocytes) confirmed that neural cells in the cortex lateralize early along the midline, while microglia originate from distinct origins. At the forebrain level, the hippocampus exhibited more clonal restriction than the neocortex or basal ganglia, implying early divergence^2^.

**Fig. 3:**
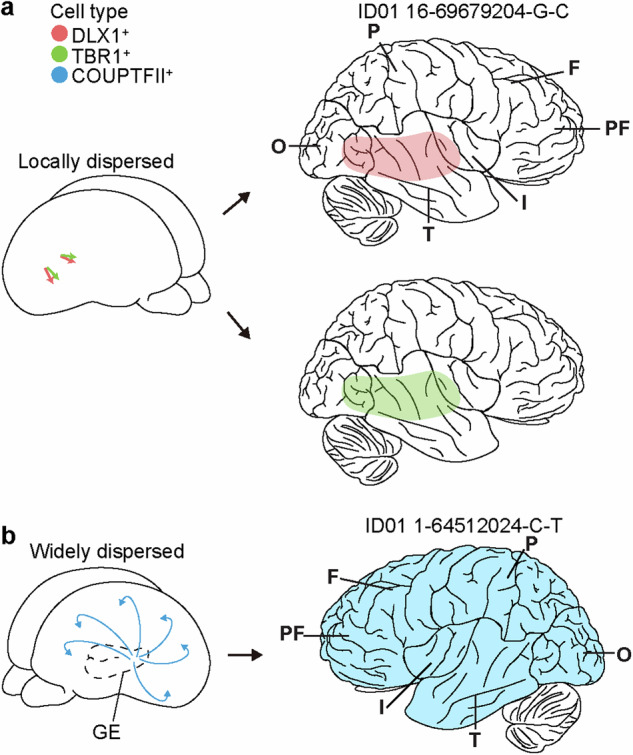
cMVBA infers dorsally-derived cortical inhibitory neurons in the human cortex. **a** The distribution of an example MV enriched within DLX1^+^ (paninhibitory neurons) and TBR1^+^ (excitatory neurons) populations but not in COUPTFII^+^ populations (CGE-derived inhibitory neurons). This distribution infers that DLX1^+^ populations may derive from similar local progenitors that produce TBR1^+^ populations and then locally disperse. **b** The distribution of an example MV enriched in COUPTFII^+^ neurons but not other cell types, which infers that COUPTFII^+^ neurons are derived from distinct origins from TBR1^+^ and DLX1^+^ populations and then widely disperse across cortical lobes. PF prefrontal, F frontal, P parietal, O occipital, T temporal, I insular, GE ganglionic eminence.

In mice, cortical inhibitory neurons dynamically migrate over long distances from the ventral telencephalon to reach the cortex^54–59^. However, a growing, sometimes conflicting, literature suggested a possible dorsal telencephalon source for inhibitory neurons in primates on the basis of marker staining or in vitro culture systems^60–65^, but direct evidence for shared lineages between excitatory and inhibitory cells in the human brain was lacking. Two recent studies addressed this question, one utilizing cMVBA, which couples FANS with multiomics, and the other utilizing cell typing and allelic fraction methods^2,6^. Intriguingly, a subset of MVs were shared between colocalized excitatory and inhibitory neurons that were not shared with cells at more distant locations, indicating that both inhibitory and excitatory neurons are produced by the same progenitor pool and locally disperse (Fig. 3). These results highlight the unique aspects of human brain development revealed through BSM analysis.

## BSM associated with focal epilepsy

Focal epilepsy is characterized by seizures originating from a specific area of the brain, known as the epileptic focus. A significant percentage of these epileptic foci exhibit dysplasia, which is often identified on neuroimaging because of their distinct structure. Given that many cases are refractory to standard antiepileptic medications, identifying the underlying mechanisms is crucial for the development of new treatments. A prominent characteristic of conditions such as hemimegalencephaly (HME) and FCD is the local distribution of dysplastic or ‘balloon’ cells within the lesion, referred to as focal malformations of cortical development (fMCD)^66^.

Approximately 30 to 70% of such resected lesions display pathogenic MVs, which are absent in peripheral tissues^67–69^. Some of the proposed explanations accounting for this variability are the differences in allelic fraction, ascertainment criteria and the proximity of the sequenced tissue to mosaic cells. Most genes identified to date are in the mTOR pathway, especially in lesions termed Type II FCD and HME^70–75^. In contrast, SLC35A2 loss-of-function mutations are primarily found in ‘mild malformation of cortical development with oligodendroglial hyperplasia and epilepsy (MOGHE)’^76^. Extensive cohort studies have clarified the genetic landscape of fMCD^14,68,77^, identifying additional genes within the mTOR or MAPK pathways and pinpointing genes linked to synaptic function, calcium dynamics, and regulatory mechanisms of gene expression in the nucleus^14^. Certain clinical phenotypes are associated with genetic clusters, suggesting that genotype‒phenotype correlations may emerge.

Mutations in genes of the mTOR pathway are more often found in dysmorphic neurons and balloon cells within the fMCD than in normal-appearing cells^69^, and FANS analysis of phosphorylated S6^+^ cells revealed higher MV allelic fractions than other cell types^10,75^. Moreover, single-nuclei RNA-Seq revealed that the expression levels of genes linked to fMCD are significantly greater in dysmorphic cells^14^, suggesting a functional connection of fMCD genes^78^.

Where do the mutations in fMCD arise? The transmantle sign—a funnel-shaped hyperintensity tapering toward the lateral ventricle found in type II FCD—suggests a radial glial clone origin during embryogenesis^79,80^. In other types of FCD, mutations may arise in different cell types or different locations. As mentioned above, a recent study revealed that a 1q tetrasomy in brain tissue resected from 4 independent FCD patients was derived from maternal germline cells (Fig. 1)^33^. This implies that the presence of a localized lesion does not necessarily result from late mutation acquisition. Instead, it could be attributed to prezygotic mosaicism followed by partial postzygotic rescue, mirroring phenomena observed in earlier studies on revertant mosaicism^81–83^. Investigating the spatial and temporal origins of dysplastic cells will increase our understanding of focal epilepsy associated with fMCD.

## BSM associated with neuropsychiatric disorders

Causes of early-onset neuropsychiatric conditions such as autism spectrum disorders (ASDs) remain an intense area of study, and while de novo mutations account for ~25%, most cases remain genetically unresolved^84,85^. Several studies have shown the association of MVs in either peripheral tissues or directly in postmortem brain tissues of individuals with ASDs^17,18,86,87^. Intriguingly, damaging, nonsynonymous MVs within critical exons of prenatally expressed genes are more common in ASD probands than in controls^86^. Furthermore, ASD brains exhibit MVs in enhancers active in the brain, which disrupt the expression of genes vital for brain function^18^ and potentially contributing to ASD risk (Fig. 1).

The relationship between BSM and later-onset diseases such as schizophrenia remains unclear. While some studies suggest a greater burden of somatic SNVs and indels in the brains of patients with schizophrenia^88^, others show no difference compared with controls^31^, albeit with a greater concentration of genes pivotal for neuronal activity^89^. This discrepancy may stem from an insufficient sample size. A recent comprehensive study utilizing blood-derived genotype arrays from 12,834 patients with schizophrenia and 11,648 controls revealed that somatic CNVs were significantly more prevalent in patients, even after CNVs at sites commonly mutated in clonal blood disorders were excluded^90^. Notably, recurrent deletions in the exons of *NRXN1* and *ABCB11* were identified, with *ABCB11* showing high expression in neurons associated with the mesocortical and mesolimbic dopaminergic pathways, areas previously linked to schizophrenia (Fig. 1). However, since these observations were not made directly in the brain or dopaminergic neurons, the precise impact of these genes on schizophrenia phenotypes remains uncertain.

## Innovative approaches for the detection of MVs

Low- or even ultralow-level MVs have the potential to be pathogenic in the brain if they impact critical signaling pathways^91,92^. However, in the human brain, where various cell lineages coexist and most cells are postmitotic and stationary, normal alleles can overshadow mutant alleles. Additionally, the difficulty of obtaining human brain samples has historically hindered progress in the BSM field. The landscape is evolving due to advancements in methodologies, which have improved the sensitivity and specificity of mutation detection (Table 1).

**Table 1:** Summary of recent innovative MV detection approaches.

Type	Potential for clinical application	Method	Result	Ref
Bulk	Low-cost high precision mutation detection	Double sequencing	High precision, compatible with low quality DNA	^11^
Bulk	Low-cost mutation validation and allelic fraction quantification	MIPP-Seq	- Reduced effect of artifacts and amplification bias even with low input DNA ( ~ 25 ng) - Can detect allelic fractions below 1%	^93^
Bulk	Costly and requires randomized tags	Duplex sequencing	- Filtering of PCR replication errors since mutations must be present in >90% of the family of reads - High precision	^7,94,95^
Liquid (CSF)	CSF extracted to track tumor DNA concentration	Cell free DNA from CSF	- CSF samples are enriched with cell-free tumor DNA and can allow for detection of mutations specific to CNS - cfDNA yield is typically low from CSF	^13,102,103^
Bulk	Precise understanding of genetic profiles at epileptogenic foci before surgery	DNA from stereo-EEG electrodes	- Direct access to brain MVs without surgery - Can detect brain MVs not detectable in peripheral tissues or CSF - Limited input DNA level	^12,104–106^
Sorted (FACS)	MV detection rate may vary depending on the marker and sorting strategy	pS6^+^ cell by FACS	Can detect variants with very low allele fractions ( ~ 0.2%)	^10^
Single cell	Not cost-efficient	PRDD-Seq (RNA+genotype)	scRNA-Seq cell type landscape to analyze lineage and age of neurons	^53^
Single cell	Limited to certain genomic regions	Single-cell RNA/ATAC-Seq	Multi-omic analysis with large number of cells	^4,6,97^
Single cell	Very costly	Primary template-directed amplification (PTA)	Precise, uniform detection of variants at the single cell level	^2^^,8,15,28^

A significant breakthrough is the introduction of a novel pipeline designed for accurately identifying MVs through deep sequencing of technical replicates^11^. This method has proven to be highly precise, achieving a record of near-zero false-positives, even when analyzing formalin-fixed paraffin-embedded (FFPE) brain samples known for their formaldehyde-compromised, low-quality DNA. Another innovative technique, multiple independent primer PCR sequencing (MIPP-Seq), involves the use of multiple UMI-barcoded primers to pinpoint MVs with allele fractions as low as 0.025% for SNVs and indels^93^. This strategy allows for the simultaneous evaluation of hundreds of variants, significantly increasing the efficiency. In addition, duplexed bulk sequencing, which sequences multiple amplicons from both DNA strands by tracing the amplicons with unique barcodes, considerably reduces the number of errors introduced during library preparation or sequencing^94,95^.

Within the clinical setting, especially for preoperative evaluation in individuals with drug-resistant epilepsy, analyzing cell-free DNA (cfDNA) in cerebrospinal fluid (CSF) has identified MVs with a detection success rate of 25%^13^. Analysis of miniscule amounts of DNA derived from depth electrodes used in presurgical evaluation could pinpoint the limits of mutated cells to refine surgical boundaries^12^. Progress in detecting ultralow MVs in resected brain tissues has been achieved by isolating mutation-bearing cells from archived frozen brain tissues through fluorescence-activated ‘cell’ sorting (FACS)^10^. This method prevents cryodamage during tissue thawing, preserving the cell membrane and protein integrity so that pS6, a marker of mutated cells in fMCD, can be used to sort them.

Advances are not limited to bulk sequencing; single-cell MV methodologies such as PRDD-Seq and ResolveOME^96^ can interrogate both genotype (from DNA) and cell type (from RNA) concurrently within single cells^53^. Ongoing efforts to identify MVs using the 10x Genomics Chromium platform for scRNA-Seq have thus far been restricted to select genomic regions^6,97^. Methods such as template-directed amplification (PTA) can now achieve coverage of over 95% of the genome of single cells^8,15,28^. However, these single-cell techniques require further optimization to overcome limitations before they are widely adopted.

These approaches incorporated with state-of-the-art low-level MV callers such as MosaicForecast^98^, DeepMosaic^99^, single-cell genotypers such as SCAN2^8^, or combinations of multiple existing callers for best practices^100,101^ can be applied synergistically for detecting MVs.

## Conclusions

BSM plays a critical role in enhancing genetic diversity, mirroring the development and aging processes of the human brain across the lifespan. Unraveling the significance of MVs found in human brain cell genomes offers profound insights into the molecular dynamics that the brain undergoes throughout life. Furthermore, examining the spatial distribution of these mutations across different brain regions or cell types reveals key information about unique aspects of human neurodevelopment through the examination of preserved postmortem brains. MVs are linked to a variety of brain health issues, including a spectrum of pediatric neurological conditions such as focal epilepsy, psychiatric disorders, aging and neurodegenerative diseases. Conducting further functional validation studies will illuminate the direct links between MVs and these brain conditions. Advances in detecting MVs have increased our ability to pinpoint mutations with lower allele fractions more precisely and with smaller tissue samples. Combining genomic data with a range of multiomic and spatial omics techniques at the single-cell level will provide us with deeper insights into the origins and effects of MVs in the brain.
